# Altered theta oscillations in resting EEG of fibromyalgia syndrome patients

**DOI:** 10.1002/ejp.1076

**Published:** 2017-07-31

**Authors:** N. Fallon, Y. Chiu, T. Nurmikko, A. Stancak

**Affiliations:** ^1^ Department Psychological Sciences Institute of Psychology, Health, and Society, University of Liverpool UK; ^2^ Wirral University Teaching Hospital NHS Foundation Trust UK; ^3^ Pain Research Institute Institute of Ageing and Chronic Disease, University of Liverpool UK; ^4^ The Walton Centre NHS Foundation Trust Liverpool UK

## Abstract

**Background:**

Fibromyalgia syndrome (FM) is a chronic pain disorder characterized by widespread pain, sleep disturbance, fatigue and cognitive/affective symptoms. Functional imaging studies have revealed that FM and other chronic pain syndromes can affect resting brain activity. This study utilized electroencephalographic (EEG) recordings to investigate the relative power of ongoing oscillatory activity in the resting brain.

**Methods:**

A 64‐channel EEG was recorded at rest in 19 female FM patients and 18 healthy, age‐matched, control subjects. The Manual Tender Point Scale (MTPS) examination was performed to quantify tonic pain and tenderness on the day of testing along with measures of mood, arousal and fatigue. Oscillations in delta, theta, alpha, beta and gamma frequency bands were analysed using Standardised Low‐Resolution Brain Electromagnetic Tomography to evaluate sources of spectral activity throughout the whole brain.

**Results:**

FM patients exhibited greater pain, tiredness and tension on the day of testing relative to healthy control participants and augmented theta activity in prefrontal and anterior cingulate cortices. No significant differences were seen in other frequency bands. Augmented frontal theta activity in FM patients significantly correlated with measures of tenderness and mean tiredness scores.

**Conclusions:**

The findings indicate that alterations to resting‐state oscillatory activity may relate to ongoing tonic pain and fatigue in FM, and manifest in brain regions relevant for cognitive‐attentional aspects of pain processing and endogenous pain inhibition. Enhanced low‐frequency oscillations were previously seen in FM and other chronic pain syndromes, and may relate to pathophysiological mechanisms for ongoing pain such as thalamocortical dysrhythmia.

**Significance:**

Increased prefrontal theta activity may contribute to persistent pain in fibromyalgia or represent the outcome of prolonged symptoms. The findings point to the potential for therapeutic interventions aimed at normalizing neural oscillations, while further research utilizing quantitative analysis of resting EEG could benefit our understanding of fibromyalgia pathophysiology.

## 1 Introduction

Fibromyalgia syndrome (FM) is characterized by widespread chronic pain and tenderness, psychological distress and fatigue (Wolfe et al., 1990; Bennett et al., 2007). The pathophysiology of FM is not known, but functional imaging studies reveal augmented brain responses to experimental pain in patients (Gracely et al., 2002) leading to the hypothesis that FM may be due to central sensitization of pain processing (Clauw, 2014). However, experimental pain studies cannot tell us about persistent tonic pain and other ongoing symptoms of FM, nor reveal whether augmented pain activations are specific to acute bouts of pain, or representative of an ongoing state of sensitization. Therefore, analysis of resting‐state brain activity has potential to enhance our understanding of the mechanisms underlying ongoing symptoms in FM.

Electroencephalographic (EEG) recordings can be used to investigate ongoing brain activity by analysing the spectral power of brain oscillations. Stern and colleagues (Sarnthein et al., 2006; Stern et al., 2006) reported enhanced resting EEG power in theta and beta frequency bands in chronic neuropathic pain patients localized to anterior cingulate, prefrontal and somatosensory cortices. Low‐frequency spectral power increases in resting brain EEG were also reported in migraine and complex regional pain syndrome patients (Bjork et al., 2009; Walton et al., 2010), paraplegic neuropathic pain patients during imaginary movement (Vuckovic et al., 2014) and spinal cord injury patients with chronic pain (Jensen et al., 2013). This accumulation of findings was previously utilized to tentatively point towards the relevance of increased resting theta activity for broader aspects of chronic pain (Pinheiro et al., 2016).

Few studies have analysed resting EEG data in FM, which actually pointed to reduced power in low‐frequency (delta) bands (Hargrove et al., 2010; Gonzalez‐Roldan et al., 2016), and increased power in beta band which was localized to right insula and mid‐cingulate cortex (Gonzalez‐Roldan et al., 2016). However, spectral analysis of magnetoencephalographic (MEG) data from FM patients did reveal increases in theta, beta and gamma bands localized to the left dorsolateral prefrontal and orbitofrontal cortices (Lim et al., 2016). Therefore, there are inconsistencies in findings of altered resting oscillatory activity which reflect the variability seen in structural brain changes in FM (reviewed in Cagnie et al., 2014). Further research will allow for the possibility of meta‐analyses to elicit the most robust resting‐state EEG alterations for FM.

Changes in resting brain oscillatory activity may be indicative of central pathophysiology. Thalamocortical dysrhythmia (TCD), a resonant interaction between thalamus and cortex activity elicited by low‐threshold calcium spike bursts in the thalamus (Llinas et al., 1999), is characterized by increased power at low frequencies and was previously proposed as a possible cause or contributing factor for neuropathic pain and other disorders (Sarnthein et al., 2006; Stern et al., 2006; Jensen et al., 2013; Vuckovic et al., 2014) including FM (Lim et al., 2016). TCD may contribute to augmented activation of cortical pain processing regions, leading to the perpetuation and exacerbation of chronic pain (Henderson et al., 2013), and normalization of TCD could be of clinical relevance for the treatment of chronic pain syndromes (Stern et al., 2006).

This study aims to expand upon prior research of ongoing brain oscillations in FM. We investigated localized alterations to brain oscillatory activity in FM patients and age‐matched, healthy participants, and included additional measures of pain and tenderness, fatigue, arousal and mood. We hypothesized that patients would exhibit significant alterations to EEG oscillatory activity, particularly in low‐frequency bands, which would localize to generators in brain regions relevant for pain processing.

## 2 Methods

### 2.1 Participants

Nineteen female patients (age 40.0 ± 8.0 years, mean ± SD) diagnosed with FM took part in the study. Patients were recruited from outpatient fibromyalgia clinics at two regional National Health Service Foundation Trust hospitals; the Walton Centre, Liverpool, United Kingdom, and Wirral University Teaching Hospital, Wirral, United Kingdom. All patients fulfilled American College of Rheumatology criteria for diagnosis with fibromyalgia (Wolfe et al., 1990), and those with additional disease or disorders which are not commonly comorbid with FM were excluded. The precise recruitment, medication and symptom profiles for the patient population can be found in our previous report of event‐related potential differences during observed pain in FM patients (Fallon et al., 2015). Patients were aged between 19 and 52 years; mean duration of symptoms was 9.6 ± 6.9 years. Eighteen female controls (age 39.2 ± 8.0 years, mean ± SD) were recruited through Internet and campus advertisements. Volunteers were age‐matched to FM patients and those taking regular medication or currently diagnosed with any disease or disorder were excluded. All patients and volunteers were compensated for time and travel expenses. Written informed consent was obtained from all participants in accordance with the Declaration of Helsinki. The study was approved by the National Research Ethics Committee of the United Kingdom and the Research Governance Committees of both NHS Foundation Trust hospitals.

### 2.2 Procedure

Participants were accompanied to the Sensory‐Motor Laboratory in the Walton Centre NHS Foundation Trust where they underwent electrode preparation seated in a comfortable chair. The experiment consisted of an EEG recording lasting 520 s, 12 auditory beep stimuli were delivered at intervals of between 35 and 45 s (mean onset 40 s) at 60 dB sound pressure level using LabTec Spin‐50 stereophonic speakers (Logitech, Inc., Morges, Switzerland) placed 0.8 m from the subject. Participants were given standardized instructions in line with methodological recommendations for resting EEG (van Diessen et al., 2015). They were requested to relax with eyes closed while remaining awake for the duration of the experiment. However, in order to combat drowsiness, which could be profound in patients, participants were also instructed to respond to the auditory stimulus by pressing the left button of a computer mouse placed in their right hand which rested on an adjustable table supporting their right arm at a comfortable height. They received specific instructions that the main aim of the experiment was to investigate resting brain activity, and therefore not to actively focus attention towards the task.

This method was employed to reduce the progression of drowsiness during the resting period which is known to affect slow wave EEG activity (De Gennaro et al., 2000), particularly in the FM patient group who commonly suffer from fatigue. Stimulus‐response epochs from 2 s before onset of auditory stimuli until 2 s after button responses were excluded as confounds to leave residual data appropriate for resting‐state analysis.

The Activation‐Deactivation Adjective Check list (AD‐ACL) questionnaire (Thayer, 1978) was administered before and after each recording to evaluate changes in levels of energy, calmness, tension and tiredness in patients and healthy participants as a result of the rest period. Following EEG recordings, each participant underwent a clinical MTPS examination (Wolfe et al., 1990) to evaluate pain and tenderness in 18 anatomically standard FMS tender points. They also completed a series of questionnaires incorporating the Beck Depression Inventory (BDI, Beck et al., 1961), Pain Catastrophising Scale (PCS, Sullivan et al., 1995) and the Fibromyalgia Impact Questionnaire (FIQ, Burckhardt et al., 1991). Subjective ratings for subscales of the AD‐ACL were analysed using 2 × 2 mixed ANOVA to compare group (FM vs. healthy controls) and time (before vs. after EEG recording) effects of rest on mood. Mean scores for all other scales were compared using independent samples *t*‐tests. All tests were performed in SPSS v.21 software (SPSS Inc, Chicago, IL, USA). Bootstrapping method (1000 samples) was utilized for *t*‐tests and a 95% confidence interval was employed throughout.

### 2.3 EEG recordings

EEG data was recorded using a 64 channel Biosemi Ag‐ACl active‐two electrode system (Biosemi B.V, Amsterdam, Netherlands). Electrode positions were allocated according to the extended 10–20 system with respect to anatomical landmarks; pre‐auricular points and the nasion. Two bipolar, flat Ag‐ACl external reference electrodes were attached to the mastoid processes. The recording bandpass filter was 0.16–100 Hz, and the sampling rate was 512 Hz.

### 2.4 EEG data analysis

EEG data was first exported to Matlab v.8.10 (The Mathworks Inc, Natick, MA, USA) for pre‐processing utilizing EEGLAB toolbox (Delorme and Makeig, 2004). Stimulus‐response epochs from 2 s before onset of auditory stimuli until 2 s after responses were removed using an in‐house programme and residual data were visually inspected for the presence of movement or muscle artefacts. One second epochs contaminated with artefacts were excluded from analyses. The experimenter was blind to participant grouping when excluding artefacts from EEG data. The mean amount of data remaining following artefact correction was 315.68 ± 43.38 s (mean ± SD) and 323.44 ± 37.27 s in patient and healthy groups respectively. An independent samples *t*‐test indicates that there was no difference in the mean duration of data retained for spectral analyses in FM and healthy groups (*t*(35) = −0.58, *p* = 0.56).

Standardized low‐resolution brain electromagnetic tomography (sLORETA, Pascual‐Marqui, 2002) was employed to investigate the spectral power of oscillatory activity throughout the brain in each of six frequency bands: delta (1–3 Hz), theta (4–7 Hz), alpha (8–13 Hz), lower‐beta (14–22 Hz), upper‐beta (23–32 Hz) and gamma (33–48 Hz). To localize the cortical regions demonstrating alterations in oscillatory activity in FM patients, independent samples *t*‐test analysis were performed using the statistics module of sLORETA to compare mean spectral power in each group, for each frequency band across 6239 voxels (5 mm^3^) covering the mantle of the brain. A 95% confidence level was employed throughout, and permutation analysis with 5000 randomizations was utilized to correct for the performance of multiple tests across all voxels. Mean spectral power from clusters of voxels demonstrating significant differences between the FM patient and control groups were exported for each subject and frequency band. Spearman's correlation analysis was conducted to evaluate the relationship between clinical symptoms and spectral power in FM patient and control groups using SPSS (1000 Bootstrap samples, confidence interval 95%).

Finally, mean absolute band power was analysed in scalp electrode data for frequency bands which demonstrated a significant group difference at the source level. To evaluate differences between FMS patients and healthy controls, a Student's independent *t*‐test was computed for each electrode in each frequency band identified from source analysis utilizing a 95% confidence level permutation analysis with 2000 randomizations (Maris and Oostenveld, 2007) to correct for multiple tests required by 64 electrodes.

## 3 Results

Table 1 shows clinical and demographic data collected for FM patient and healthy control groups. Independent samples *t*‐tests indicate greater scores in FM patients, relative to healthy participants, for pain and tenderness on the day of testing as measured by the MTPS score, increased pain catastrophizing according to PCS and lower mood indexed by BDI. ANOVA analysis of pre‐post AD‐ACL scores revealed that FM patients were significantly more tired (*F*(35) = 8.95, *p* = 0.005) and tense (*F*(35) = 6.84, *p* = 0.013) than healthy participants, but no differences were found between for pre‐post testing changes in energy, tiredness, tension or calmness, nor was there any interaction between FM status and pre‐post changes on any scale (*p* > 0.05).

**Table 1:** Demographic and clinical data for FMS patients and healthy control participants

	FMS	Healthy	*T*	*p*
	Mean ± SD	Mean ± SD		
Age	40.01 ± 7.94	39.22 ± 7.98	0.30	0.77
FIQ	60.60 ± 17.88	5.91 ± 6.27	12.54	0.001
MTPS	4.95 ± 1.91	0.23 ± 0.32	10.93	0.001
BDI	18.79 ± 10.75	3.67 ± 4.17	5.66	0.001
PCS	14.47 ± 10.25	4.67 ± 5.97	3.34	0.007

AD‐ACL			*F*	*p*
Energetic‐Pre	1.37 ± 0.63	1.84 ± 0.80	1.83	0.185
Post	1.40 ± 0.62	1.42 ± 0.69		
Tiredness‐Pre	2.68 ± 0.82	1.97 ± 0.91	8.95	0.005
Post	2.72 ± 0.76	2.01 ± 0.62		
Tension‐Pre	1.75 ± 0.86	1.23 ± 0.32	6.84	0.013
Post	1.85 ± 1.02	1.28 ± 0.45		
Calmness‐Pre	3.32 ± 0.73	3.58 ± 0.38	2.12	0.155
Post	3.56 ± 0.58	3.72 ± 0.56		

FIQ, Fibromyalgia Impact Questionnaire; MTPS, Manual Tender Point Scale; BDI, Beck Depression Inventory; PCS, Pain Catastrophising Scale; AD‐ACL, Activation‐Deactivation Adjective Checklist; SD, Standard deviation.

**Figure 1:**
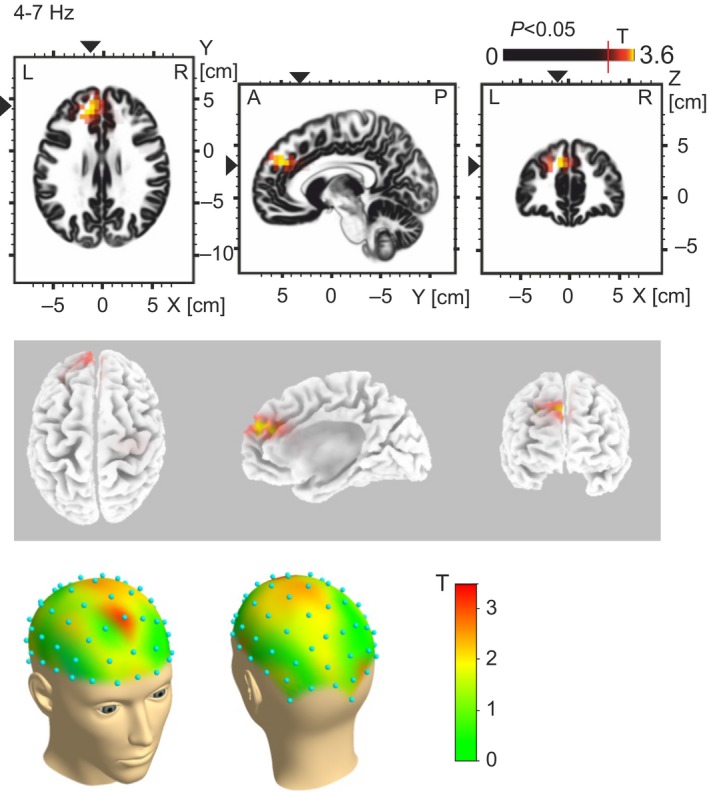
The location of augmented theta band (4–7 Hz) oscillatory activity in the FM patient group relative to healthy control participants are shown in three orthogonal planes (horizontal, sagittal, coronal, *top panel*), and three‐dimensional brains (*middle Panel*). Enhanced theta activity in FMS patients was also evident in the fronto‐central scalp electrodes (*bottom panel*).

Evaluation of sLORETA comparisons of spectral power throughout the cortical mantle in each discreet frequency band indicated that FM patients demonstrate augmented theta activity relative to healthy control participants (*t*(35) = 3.58, *p* = 0.002) in prefrontal cortex. The peak difference was located in medial prefrontal cortex (mPFC, MNI coordinates; *x* = −3 mm, *y* = 42 mm, *z* = 30 mm), but the bulk of the region demonstrating augmented theta oscillations covered voxels in the vicinity of the anterior cingulate cortex (ACC, *x* = −8 mm, *y* = 35 mm, *z* = 24 mm). The cluster also extended laterally to infringe on the dorsolateral prefrontal cortex (DLPFC, *x* = −20 mm, *y* = 45 mm, *z* = 36 mm). Anatomical locations are according to the digitized probability atlas provided by the Brain Imaging Center, Montreal Neurological Institute (Lancaster et al., 1997, 2000) incorporated in sLORETA analysis. The sLORETA maps of the difference in theta band power between patients and healthy controls are shown in Fig. 1.

Analysis of oscillatory data from scalp electrodes also revealed enhanced theta activity in FM patients with a peak in fronto‐central electrodes. Fig. 1 illustrates the location and distribution of augmented theta activity in the FM patient group. No significant differences were seen in delta, alpha, beta or gamma frequency bands.

**Figure 2:**
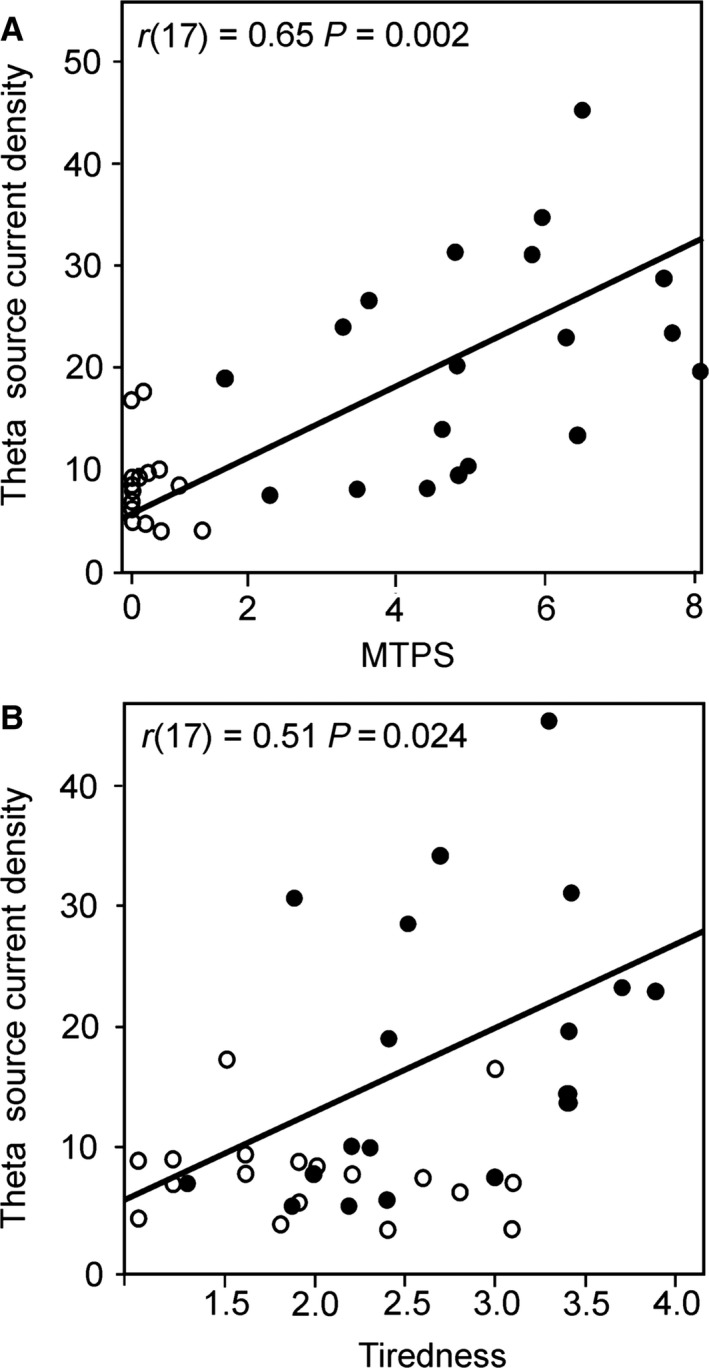
(A) Scatterplot illustrating the significant correlation between theta oscillatory band activity in frontal cortices (from cluster which demonstrated a significant difference in patients relative to healthy group) and MTPS pain scores from the day of testing in the FM patient group (solid circles) and healthy participants (transparent circles), and (B) Correlation between theta oscillatory activity and AD‐ACL tiredness scores.

Spearman's correlation analysis indicated that frontal theta activity in FM patients correlated with measures of pain and tenderness on the day of testing indexed by MTPS scores (*r*(17) = 0.65, *p* = 0.002), and also with mean (average of pre and post recording) tiredness scores measured using the tiredness scale of the AD‐ACL questionnaire (*r*(17) = 0.51, *p* = 0.024). Furthermore, mean tiredness and MTPS scores were also found to be correlated in patients (*r*(17) = 0.59, *p* = 0.008). Neither correlation was significant in healthy participants (*p* > 0.05). Fig. 2 illustrates the results of Spearman's correlation analysis between individual frontal theta band activity in the medial frontal gyrus in FM patients and healthy participants with MTPS and tiredness scores.

## 4 Discussion

Our findings indicate that FM patients exhibit increases in frontal theta power during resting EEG. Augmented low‐frequency oscillations were localized to anterior cingulate, medial prefrontal and dorsolateral prefrontal cortices. Furthermore, subjective power of low‐frequency oscillatory activity was associated with increased tenderness and tiredness scores in patients, which points to potential relevance for resting‐state electrophysiology in the manifestation of clinical symptoms of FM.

The present findings correspond with a recent MEG study of resting oscillatory activity which also demonstrated enhanced frontal theta oscillations in FM patients (Lim et al., 2016). However, it is worth noting that our results do not correspond with the recent EEG study of resting‐spectral power in FM oscillatory activity which instead pointed to reduced delta activity in right insula and enhanced beta activity in right middle frontal lobe (Gonzalez‐Roldan et al., 2016). Despite these differences, it is important to consider the possibility that findings from FM populations may reflect the heterogeneity of symptoms in a patient cohort which can influence the discernibility of neurophysiological findings (May, 2011). An initial comparison of patient profiles reveals greater BDI scores and a larger proportion of patients using antidepressant medication in the previous study, and the authors acknowledge that these factors could have some impact on electrophysiology (Gonzalez‐Roldan et al., 2016). The average age and durations of symptoms was also lower in this study which may also explain the differences in resting‐state findings.

Enhanced theta oscillations seen in FM patients in this study were localized to frontal regions including mPFC, DLPFC and ACC, and also evident in fronto‐central regions of scalp electrodes. These loci are consistent with the aforementioned MEG research (Lim et al., 2016), and also with a resting‐state fMRI study which pointed to increased spectral power at low frequencies in FM patients (Kim et al., 2013). ACC and mPFC play an important role in endogenous pain modulation (Bingel et al., 2006), and augmented theta oscillations in these regions could relate to a down‐regulation of pain inhibition (Henderson et al., 2013). Alternatively, pain‐related activation in the DLPFC has been linked to the cognitive dimension of pain, e.g. attention, localization and encoding of sensation (Peyron et al., 2000), and increased activity in DLPFC during experimental pain demonstrates a significant association with pain catastrophizing in patients with FM (Gracely et al., 2004). Therefore, enhanced theta activity in this region could also have relevance for augmented cognitive or attentional processing for ongoing pain in FM. Interestingly, anticipation for pain was also shown to influence pain report in FM, and this was again related to activation in DLPFC (Brown et al., 2014), pointing to a potential top‐down modulatory role for DLPFC in the ongoing pain experience of patients.

Increases in low‐frequency resting EEG power were previously proposed to relate to TCD which may represent a pathophysiological model for chronic pain across multiple disorders including FM (Llinas et al., 1999; Lim et al., 2016). Thalamic relay neurons directly influence the oscillatory activity of thalamocortical loops (Sarnthein and Jeanmonod, 2008), and the frontal brain regions highlighted by the present findings receive projections from the medio‐dorsal nucleus of the thalamus (Klein et al., 2010). Therefore, TCD represents one plausible explanation for augmented theta activity in frontal cortices seen in this study and previous research of other chronic pain populations (Sarnthein et al., 2006; Stern et al., 2006; Walton et al., 2010). Researchers have demonstrated structural (Schmidt‐Wilcke et al., 2007; Lutz et al., 2008) and functional (Gracely et al., 2002; Jensen et al., 2009, 2012) alterations in the thalami of FM patients, and similar changes were proposed as evidence of TCD in chronic neuropathic pain (Henderson et al., 2013). Furthermore, a mechanism for the role of TCD in persistent pain was previously proposed, suggesting that enhanced frontal theta activity is indicative of reduced inhibitory capacity for endogenous pain modulation which in turn contributes to the overall perception of persistent pain (Henderson et al., 2013).

Further research is needed to evaluate whether TCD represents a valid potential pathophysiological mechanism for FM, and whether the differences seen reflect a predisposition for chronic pain or a maladaptive change associated with years of ongoing symptoms. However, if TCD can be shown to play an active role in the experience of FM and other syndromes, then this could have important clinical implications. For example, frontal theta power in neuropathic pain patients was shown to normalize, aligned with improvement in pain symptoms, following therapeutic lesions of the thalamus (Sarnthein et al., 2006), and peak alpha and theta frequencies were recently utilized as a marker of therapeutic efficacy for Transcranial Direct Current Stimulation in neuropathic pain (Ngernyam et al., 2015). Augmented theta activity in this study was also evident in scalp data over fronto‐central electrodes, which highlights the potential for clinical evaluations of theta which may require only basic clinical‐EEG and minimal processing.

In addition to FM, it is also worth highlighting that similar patterns of enhanced frontal theta activity were previously reported in neuropathic pain (Sarnthein et al., 2006; Stern et al., 2006; Jensen et al., 2013; Vuckovic et al., 2014), complex regional pain syndrome (Walton et al., 2010) and migraine patients (Bjork et al., 2009). It is feasible that enhanced frontal theta power relates to chronic pain in a broader sense, and this proposition was tentatively alluded to in a recent systematic review of electrophysiological studies (Pinheiro et al., 2016). This leads to the possibility that oscillatory changes seen in various chronic pain syndromes could relate to a common mechanism of pathophysiology, or represent a consequence of prolonged ongoing pain or associated symptoms. In this study, the relationship between augmented theta and tiredness indicates that fatigue could potentially contribute to low‐frequency oscillatory changes. Fatigue is a common symptom among many chronic pain syndromes, and its effects on brain electrophysiology warrant further research. However, any potential common relationship between resting brain activity and broader aspects of chronic pain are speculative and require further investigation. It is also worth noting that this body of research is primarily populated with findings from neuropathic pain patients.

This study has some obvious limitations. As mentioned, the cross‐sectional design means that it is impossible to infer whether the changes seen represent a predisposing pathophysiological cause of FM symptoms, or a consequence of years of chronic pain and other symptoms such as fatigue. This is further complicated by the existence of a clear relationship between these symptoms in patients, and the role of sleep deprivation in maintaining pain symptoms is a focus of discussion for FM (Choy, 2015). Secondly, in the absence of other patient group comparisons, it is not possible to infer whether the difference seen relate specifically to FM or more general aspects of chronic pain. However, our finding of enhanced frontal theta does correspond with findings from multiple chronic pain syndromes (Sarnthein et al., 2006; Stern et al., 2006; Bjork et al., 2009; Walton et al., 2010; Jensen et al., 2013; Vuckovic et al., 2014) which highlights the need for further research of this phenomenon. Although we reveal alterations in cortical rhythms localized to frontal cortices, any relationship with potential pathophysiology such as TCD is speculative in the absence of, for example, direct recordings of thalamic activity which would confirm abnormal thalamocortical rhythms.

Measures were taken to ensure participants remained awake and alert throughout recordings, and stringent levels of control were applied during recruitment. However, it is still possible that low‐level medications permitted in the study, or other confounding factors such as drowsiness, could unduly influence findings. Alternatively, the methodology employed to restrict drowsiness could have some influence on EEG signals, and the auditory task could unduly affect patients who may exhibit hypervigilance to their surroundings. However, EEG studies using spectral analysis have shown a high degree of similarity between resting and ‘task waiting’ data (Fingelkurts et al., 2006), and augmented auditory processing in FM patients was previously shown to be restricted to reduced noxious thresholds for loud intensity stimuli (Carrillo‐de‐la‐Pena et al., 2006). Likewise, research comparing EEG data for processing of painful and moderate auditory stimuli in FM patients indicated that hypervigilance for low‐level auditory stimuli, as utilized in this study, did not occur (Lorenz et al., 1996). Although our findings correspond to low‐frequency changes seen in FM patients using MEG (Lim et al., 2016), we do not find alterations in higher frequency bands, and recent EEG studies of resting‐spectral power in FM oscillatory activity did not reveal augmented low‐frequency oscillations (Gonzalez‐Roldan et al., 2016). However, variability is commonplace in studies of FM, which may be due to heterogeneity of symptoms and treatments in each patient cohort (May, 2011). Perhaps, the most robust findings in FM will only emerge from many studies leading to meta‐analysis or systematic reviews.

To conclude, FM patients exhibited enhanced theta oscillatory activity in brain regions which is important for endogenous pain inhibition and attentional‐cognitive aspects of pain processing. The alterations show a relationship with ongoing symptoms including pain and tiredness indicating that increased prefrontal theta activity may contribute to persistent perception of pain in FM or represent the outcome of years of pain and fatigue. The findings suggest that therapeutic interventions aimed at normalizing neural oscillations may lead to some relief of ongoing symptoms for patients, or reveal more about FM pathophysiology. However, further research is needed to clarify the precise nature and time course of oscillatory changes in FM.

## Author contributions

NF takes responsibility for the integrity and accuracy of the data. AS and TN conceived and designed the study. NF collected the data, and NF and AS performed the data analyses. NF drafted the manuscript. All authors discussed the results and commented on the manuscript and approved the final version.
